# Audiogenic epileptic DBA/2 mice strain as a model of genetic reflex seizures and SUDEP

**DOI:** 10.3389/fneur.2023.1223074

**Published:** 2023-08-23

**Authors:** Francesca Bosco, Lorenza Guarnieri, Antonio Leo, Martina Tallarico, Luca Gallelli, Vincenzo Rania, Rita Citraro, Giovambattista De Sarro

**Affiliations:** ^1^Section of Pharmacology, Science of Health Department, School of Medicine, University “Magna Graecia” of Catanzaro, Catanzaro, Italy; ^2^Research Center FAS@UMG, Department of Health Science, University “Magna Graecia” of Catanzaro, Catanzaro, Italy

**Keywords:** generalized tonic–clonic seizure, sudden unexpected death in epilepsy, adenosine and serotonin neurotransmission, audiogenic seizures, DBA/2 mice, cardiorespiratory dysfunction

## Abstract

Epilepsy is a chronic neurological disease characterized by abnormal brain activity, which results in repeated spontaneous seizures. Sudden unexpected death in epilepsy (SUDEP) is the leading cause of seizure-related premature death, particularly in drug-resistant epilepsy patients. The etiology of SUDEP is a structural injury to the brain that is not fully understood, but it is frequently associated with poorly controlled and repeated generalized tonic–clonic seizures (GTCSs) that cause cardiorespiratory and autonomic dysfunctions, indicating the involvement of the brainstem. Both respiratory and cardiac abnormalities have been observed in SUDEP, but not much progress has been made in their prevention. Owing to the complexity of SUDEP, experimental animal models have been used to investigate cardiac and/or respiratory dysregulation due to or associated with epileptic seizures that may contribute to death in humans. Numerous rodent models, especially mouse models, have been developed to better understand epilepsy and SUDEP physiopathology. This review synthesizes the current knowledge about dilute brown agouti coat color (DBA/2) mice as a possible SUDEP model because respiratory arrest (RA) and sudden death induced by audiogenic generalized seizures (AGSs) have been observed in these animals. Respiratory/cardiac dysfunction, brainstem arousal system dysfunction, and alteration of the neurotransmitter systems, which are observed in human SUDEP, have also been observed in these mice. In particular, serotonin (5-HT) alteration and adenosine neurotransmission appear to contribute to not only the pathophysiological mechanisms of medication but also seizure-related respiratory dysfunctions in this animal model. These neurotransmitter systems could be the relevant targets for medication development for chronic epilepsy and SUDEP prevention. We reviewed data on AGSs in DBA/2 mice and the relevance of this model of generalized tonic–clonic epilepsy to human SUDEP. Furthermore, the advantages of using this strain prone to AGSs for the identification of possible new therapeutic targets and treatment options have also been assessed.

## Introduction

Epilepsy is a chronic and debilitating neurologic disease with a high prevalence, which may substantially impair the quality of life. It is associated with cognitive decline and other neuropsychiatric comorbidities, as well as pharmacoresistance development, contributing to increased mortality (1). Sudden unexpected death in epilepsy (SUDEP) is the most frequent cause of epilepsy-related death in drug-resistant epileptic patients, and repeated generalized tonic–clonic seizures (GTCSs) are associated with SUDEP, leading to insistent brain activity damage and enhanced sympathetic activation (2). Seizure activation can propagate to distinct subcortical structures via synaptic connections or, more likely, via spreading depression, which can alter brainstem activity and impair cardiorespiratory function (3). The risk of sudden unexpected death is estimated to be 20 times higher in epileptic patients than in the general population (4). For treating SUDEP, new therapeutic treatments are needed to reduce disease progression and show efficacy against drug-resistant epilepsy, reducing the risk of SUDEP.

Given the unpredictable nature of SUDEP, it is difficult to study the mechanisms by which seizures propagate and impair brainstem function, producing the cardiorespiratory effects that induce it. Significant progress has been made in understanding the SUDEP mechanisms through clinical and experimental studies. Nevertheless, the etiology and pathogenesis of SUDEP are incompletely understood (1, 5). Different clinical and animal studies have indicated that SUDEP occurs due to multifactorial factors and that seizure-induced respiratory dysfunction, together with brainstem system dysfunction and neurotransmitter dysregulation, plays an important role in the mechanism of SUDEP in human and rodents (1, 6).

Genetic factors, such as channelopathies, or susceptibility to heat or audiogenic-induced seizures also contribute to increased predisposition to SUDEP in animal models (7). Studies on genetic mouse models allow for a better understanding of the pathophysiological modifications that lead to SUDEP (5). Usually, mouse models of sound-induced audiogenic generalized seizures (AGSs) are used to study the underlying mechanisms of SUDEP, with the aim of treating and preventing respiratory arrest (RA) due to AGSs (7). In audiogenic susceptible mice, intense auditory stimulation produces severe reflex tonic–clonic seizures that can frequently be lethal, mimicking those observed in human (8). The dilute brown agouti coat color (DBA/2) mice are a model well-established for audiogenic reflex epilepsy induced by sound stimulation, and they were used to investigate new antiseizure medications (ASMs) (9). These mice were also employed in the SUDEP model since they showed generalized convulsive seizures followed by respiratory arrest, which subsequently led to cardiac arrest and sudden death, similar to what is observed in human SUDEP (6, 10). Studies on DBA/2 mice have investigated drugs that reduce immediate RA and seizure-induced death (11). We reviewed the literature with information about DBA/2 mice as a model of SUDEP. We also reviewed the neuronal and biochemical mechanisms of reflex epilepsy and SUDEP, as well as the development of better treatment options.

## SUDEP in epilepsy

SUDEP refers to an unexpected, non-traumatic death in children and adults with epilepsy, where postmortem examination does not reveal any anatomical or toxicological cause of death, including drowning (12, 13). Among the most important risk factors for SUDEP are uncontrolled or frequent GTCSs, the duration of epilepsy, prone position at the time of death, young age of first seizure, male sex, neurological comorbidities, polytherapy, ion channel or arrhythmia-related gene mutations, cardiac-respiratory dysfunction, intellectual disability, nocturnal seizures, and non-adherence to ASMs (12, 14). Severe GTCS with a frequency of >3/year have been clarified to induce changes in autonomic functions, resulting in the impairment of respiratory and cardiac functions and predisposing to SUDEP (1).

The incidence of SUDEP is estimated to be 24 times higher in epileptic patients than in the general population, with 1.16 cases per 1,000 epileptic patients per year, and the rate is higher in patients with refractory epilepsy (15). Furthermore, SUDEP reportedly affects all age groups, and its incidence is considered to be less common in young children than in adults, excluding rare diseases such as epileptic syndromes or genetic epilepsies (16). Recently, the risk of SUDEP in children has been found to be potentially greater than that in previous years (from 0.13 to 3.3 per 1,000 patients), especially in children with an increased severity of epilepsy in terms of frequency and type of seizures. Pediatric-specific risk factors include developmental delay and intellectual disability, structural abnormalities, and multiple ASM therapy (17).

The risk of SUDEP was markedly increased in children with genetic epilepsies, Dravet syndrome due to mutations in the *SCN1A, SCN8A* encephalopathies, or *DEPDC5* gene mutation-related epilepsy (18–20). However, the incidence of SUDEP in children and young people with epilepsy remains indeterminate because it varies depending on age range, type of epilepsies, epileptic syndromes, or genetic epilepsies, as well as the follow-up period and diagnosis (18). Successive cohort studies have suggested that the incidence of SUDEP is similar between adults and children (i.e., 1.2 per 1,000 people per year), although the incidence may be underestimated in children due to non-epilepsy-related deaths, as a post-mortem/autopsy is often not performed (21). To date, the exact incidence of SUDEP within subgroups of childhood epilepsy is not known.

All the SUDEP cases died at an early age (generally 10–40 years), particularly, patients with intellectual impairment, refractory epilepsy, and poorly controlled epilepsy with a high frequency of GTCS and nocturnal seizures. Studies have suggested a slight male predominance, with the male-to-female ratio being 229:159 (13, 22).

## DBA/2 mouse

The DBA/2 inbred strain is genetically susceptible to AGSs, evoked by excessive auditory stimulation. AGSs consist of generalized reflex clonic–tonic convulsions, followed by seizure-induced respiratory arrest (S-IRA) (23). These events, observed in clinical SUDEP, make DBA/2 mice relevant models of SUDEP (23, 24). This strain has been well-described both phenotypically and genetically (9). In DBA/2 mice, the susceptibility to AGSs varies with age when exposed to intense auditory stimulation (100–120 dB), being maximal between 21 and 28 days of age and then reduced or absent at 40–45 days (25). After intense sound stimulation, DBA/2 mice show seizure sequences characterized by brainstem-dependent wild runs and jumps, followed by clonic seizures characterized by violent convulsions and muscle spasms and, subsequently, tonic seizures characterized by muscle rigidity, culminating in S-IRA, cardiac dysfunction, and death. In DBA/2 mice, death occurs following an excessive tonic phase with hindlimb extension but not from clonic seizures (7), indicating that the tonic phase is probably responsible for S-IRA. Respiratory arrest is fatal unless the death is prevented through oxygenation or mechanical ventilation (26).

In ~75% of these mice, AGSs are followed by RA, while the remaining 15% of DBA/2 mice exhibit AGSs without RA, indicating that they spontaneously recover from RA post-AGSs (25, 27). In DBA/2 with S-IRA, while RA is the main cause of death, electrocardiographic activity can be detected for ~5 min after RA, suggesting that cardiac changes occur later (28).

The severity and susceptibility of the seizure to S-IRA decrease after 5 weeks of age due to hearing loss, with hearing thresholds elevated by 15–20 dB (29). This hearing loss may be due to the loss of sensory hair cells, the loss of spiral ganglion neurons, or striatal atrophy (30, 31). Progressive hearing loss has also been attributed to mutations in Cdh23 and Fscn2 (32, 33).

DBA/2 mice are the first example of polygenic heredity in which several mutations can be related to AGSs. More specifically, three loci, including Asp1, Asp2, and Asp3, located on chromosomes 12, 4, and 7, have been correlated with AGSs (34–36). In particular, Asp1 and Asp2 loci seem to be responsible for the manifestations of AGSs (35). These loci are also involved with the regulation of Ca^2+^-ATPase activity, which is important for synaptic function and neurotransmitter release from synaptic vesicles (34).

DBA/2 mice also express an astrocyte-specific Kcnj10 deletion that has been shown to disrupt the activity of inward rectifying potassium (Kir) 4.1 channels and uptake glutamate (37); this results in low seizure threshold of audiogenic mice compared with the seizure-insensitive C57BL/6J mice (38).

Consequently, Kcnj10 gene polymorphism could play an important role in seizure-threshold differences between DBA/2 and C57BL/6J mice (37). DBA/2 mice are homozygous for allele 1473G and have a lower serotonin (5-HT) synthesis rate compared with C57BL/6J mice; this polymorphism, which alters brainstem serotonergic neurotransmission, contributes to AGSs and S-IRA in these mice (39, 40).

## Pathophysiology of audiogenic seizures in DBA/2 mice

The production of AGSs resides in the interaction of brainstem sensory-motor structures in DBA/2 mice (27). In response to intense sound stimulation, a small population of hyperexcited neurons, which are located at the level of the inferior colliculus (IC) of the midbrain, induce the initiation and propagation of AGSs (8, 41). Thus, the IC is considered to be a crucial structure for AGSs onset, but other subcortical structures such as the rostral and medial subcortical regions may be involved (42), resulting in clonic and tonic seizures. These AGSs subsequently exert a negative effect on breathing, inducing S-IRA (41, 43). Studies on DBA/2 mice show that AGSs inhibit the ponto-medullary respiratory control network responsible for breathing control, which leads to apnea or respiratory arrest followed by asystole and death (44).

A key role of the IC in AGSs in DBA/2 mice has been shown by an experimental study, in which bilateral lesions of IC either eliminated or reduced AGS, whereas spreading depression of the cortex either increased latencies or decreased the severity but did not fully abolish AGSs (41). This does not seem to be due to the interruption of sensory input to the cerebral cortex because lesions of other relay nuclei fail to block AGSs (41). Studies based on fos immunochemistry have confirmed that the onset of AGSs is due to enhanced activity within the IC (45). The superior colliculus (SC) is an important modulatory structure in the network of AGSs, and its role is supported by the incomplete attenuation of AGSs through SC lesions (41). Epileptic foci have been also found in the cortex of DBA/2 mice, suggesting an involvement of cortical activities in the generation of AGSs (46). MEMRI data have demonstrated in DBA/1 mice, another model of AGS, changes involving various subcortical structures, such as the superior olivary complex (SOC) of the brainstem, the periaqueductal gray complex, and the amygdala, during AGS. In DBA/1 mice, pathways starting from the SOC play a key role in the neuronal network involved in audiogenic seizures; similarly, in DBA/2 mice, neuronal circuits in the brainstem, spinal cord, and all subcortical areas could also be important (47, 48).

## Alteration of neurotransmissions in DBA/2 mice

The susceptibility and severity of AGSs in DBA/2 mice involved several neurotransmitters, such as serotonin (5-TH), adenosine, norepinephrine (NE), dopamine (DA), acetylcholine, glutamate, and GABA (56) (Table 1). Among these neurotransmitters, the serotonergic system is responsible for the generation and transmission of the respiratory rhythm in the brainstem (57), as well as in the modulation of epileptic seizures (58, 59). Accordingly, dysfunction in the brainstem 5-HT system, which results in impaired synaptic transmission and alterations in the expression of 5-TH receptors, is involved in respiratory dysfunction and is associated with a more severe respiratory phenotype in DBA/2 mice (25, 47). These mice generally have lower 5-HT tissue levels than C57/L6 control mice, and the lack of serotonergic signaling of raphe neurons contributes to their increased susceptibility to seizures and RA (39, 60, 61). Therefore, a reduction in 5-TH levels is believed to be responsible for increased seizure susceptibility, while the administration of its precursor 5-hydroxytryptophan (5-HTP), which increases 5-TH brain levels, can attenuate the propagation of AGSs in DBA/2 mice (62). Furthermore, 5-THP has also been found to counteract the reserpine-induced increase of AGSs in DBA/2 mice (63). Alterations of the serotonergic system may also be associated with a more severe respiratory phenotype because DBA/2 mice with fatal RA showed higher levels of tryptophan hydroxylase 2 (TPH2) and serotonin transporter (SERT) than DBA/2 mice with non-fatal RA (44). In addition, DBA/2 mice showed altered expression of 5-HT receptors in the brainstem respiratory nuclei compared with C57BL/6J mice, with diminished expression of 5-HT2C, 5-HT3, and 5-HT4 receptors in the rostral ventral medulla respiratory region and enhanced expression of 5-HT2B receptors (11, 40). The changes in the brainstem 5-HT receptor expression are consistent with the increase of excitation mediated by 5-HT2B elevated expression, and the decrease of inhibition is mediated by decreased 5-HT2C expression; these changes are associated with susceptibility to AGSs and S-IRA in DBA/2 mice (40, 64). Antagonist compounds of 5-HT1C, 5-HT3, 5-HT4, and 5-HT7 receptors had anticonvulsant effects, increasing the latency of AGSs and decreasing the severity of seizures (65, 66), while antagonist compounds of 5-HT2 receptors increased the S-IRA incidence following seizures in DBA/2 mice (25).

**Table 1:** Effects of different monoaminergic receptors on the pathogenesis of SUDEP in DBA/2 mice.

**Neurotransmitters**	**Type of effects**	**Type of receptors**	**Respiration**	**Audiogenic seizures/SUDEP**	**References**
5-HT	Lower 5-HT levels	Increased expression of 5-HT2B	–	+	(25, 47)
		Decreased expression of 5-HT2C, 5-HT3, and 5-HT4	–	+	(11, 40)
Adenosine	Increases in extracellular adenosine	Over-stimulation of adenosine A1 and A2a	–	+	(49)
Noradrenaline	Lower levels	Reduced number of α1-adrenoceptor binding sites	*n*	+	(50)
Dopamine	Lower DA levels		*n*	+	(51)
Glutamate	Increased function	Upregulation of NMDA	*n*	+	(52)
GABA	Decreased function	GABA_A_	*n*	+	(53–55)

+, activation; –, inhibition; *n*, no evidence.

Furthermore, the decrease of AGSs has been found with an inhibitor of tryptophan hydroxylase, parachlorophenylalanine (PCPA), or 5-HT receptor antagonists (66, 67). Therefore, reduced 5-TH neurotransmission due to depletion of the reserve or antagonism of 5-HT receptors are factors that may exert protection against AGSs in DBA/2 mice (40).

Owing to the effects exerted by 5-HT on respiration and seizures, it can be suggested that drugs that enhance the activity of this neurotransmitter might be useful in SUDEP prevention. Consequently, treatment with fluoxetine, a selective serotonin reuptake inhibitor (SSRI), has been found to be effective in preventing S-IRA and death, without affecting the severity of AGSs in DBA/2 mice (25).

Furthermore, the administration of fluoxetine at higher doses was able to reduce the occurrence of tonic seizures preceding the S-IRA (25). Additionally, the combined administration of serotonin uptake inhibitors and monoamine oxidase-A (MAO) inhibitors diminished AGSs in DBA/2J mice (62). On the contrary, the administration of cyproheptadine, a non-selective 5-HT receptor antagonist, increased susceptibility to S-IRA in 10% of DBA/2 mice, confirming further involvement of the serotonergic system in S-IRA (25). Therefore, genetic alterations of the 5-HT system, such as specific 5-HT receptor subtype expression abnormalities or reduced levels of the 5-HT-synthesizing enzyme tryptophan hydroxylase, can be responsible for the susceptibility to S-IRA of DBA/2 mice (40, 47).

Adenosine is another neurotransmitter that exerts its action on the respiratory centers of the brainstem, and an increase in its levels, induced by seizures, may contribute to SUDEP (68). From this neurotransmitter, that serotonin and adenosine show the opposite effects on respiratory suppression. While elevated serotonin levels in the brainstem can prevent S-RIA and death in DBA/2 mice, overstimulation of adenosine receptors in the brainstem can induce respiratory arrest (28). Some studies have shown that molecules such as caffeine, a non-selective adenosine receptor antagonist, or SCH 442416, an A2A receptor antagonist, decrease the occurrence of S-IRA in DBA/2 mice. Other studies have demonstrated that DPCPX, a selective adenosine A1 antagonist, did not alter S-IRA, suggesting that excessive A2A receptor activation makes DBA/2 mice more vulnerable to S-IRA (28, 69). The inhibition of adenosine metabolism by pretreatment with 5-iodotubercidin (5-ITU) increased the susceptibility to S-IRA of DBA/2 mice, which initially displayed only AGSs, indicating that excessive adenosine signaling may contribute to seizure-induced death in these mice (28). Reduced brain levels of NE and DA are also responsible for increased AGS susceptibility (51). Generally, DBA/2 mice show lower levels of NE and DA in the brain than C57BL/6J mice; the administration of Levodopa (L-DOPA) was found to attenuate AGS in DBA/2 mice by increasing the brain levels of these neurotransmitters, particularly DA, which is the active principle formed from L-DOPA (51, 70).

In addition to L-DOPA, dopaminergic receptor agonists, such as apomorphine and other related compounds, showed protection against AGS in DBA/2 mice (71).

Alterations in the glutamatergic and GABAergic neurotransmission are also involved in the generation of AGS in DBA/2 mice (9, 72). Regarding glutamatergic neurotransmission, an increased NMDA-mediated glutamate release has been found to occur in 21–30-day-old DBA/2 mice through a nitric oxide (NO)-mediated mechanism (73). At this age and following audiogenic stimulation, the enhanced NO generation in DBA/2 mice was 2-fold compared with DBA/2 mice, which were not exposed to acoustic stimulation, indicating the involvement of NO in the occurrence of AGS (74). The antagonists of ionotropic and metabotropic glutamatergic receptors exerted inhibitory effects on AGS, supporting the involvement of glutamatergic neurotransmission (75–78). In particular, a functional NMDA-mediated upregulation responsible for the generation of AGS has been shown in DBA/2 mice, and the administration of an antisense probe for the NMDA receptor (NR1 subunit) has been shown to induce a complete suppression of AGS, accompanied by a small (20%) reduction in the NMDA receptor levels (79).

**Figure 1:**
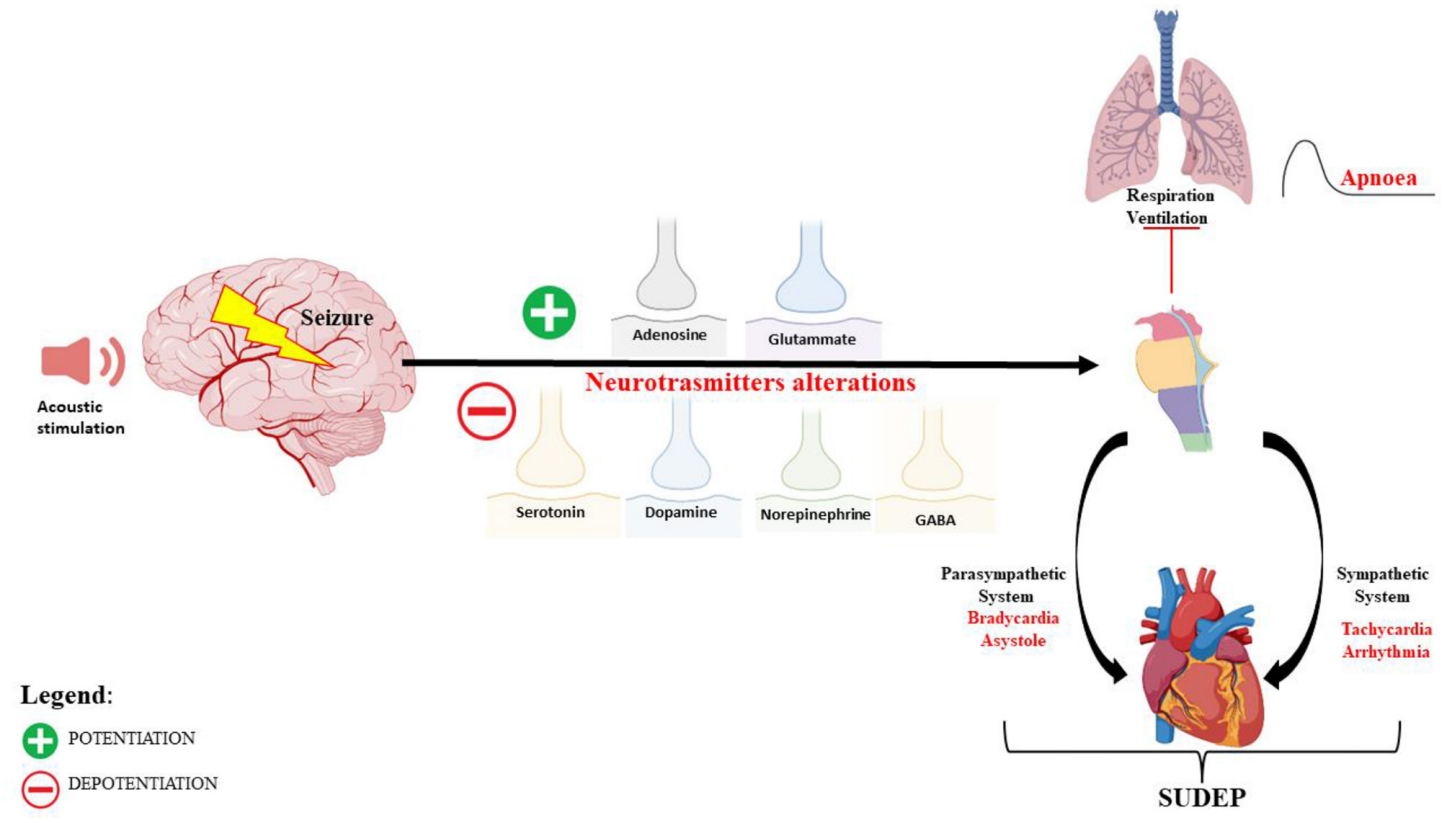
Schematic representation of the neuronal network and mechanisms of autonomic central cardiorespiratory dysfunction during seizure following audiogenic stimulation in DBA/2 mice.

A deficit of GABAergic neurotransmission, in IC neurons, is also implicated in the AGS susceptibility of DBA/2 mice (80), and drugs that increase GABA concentration or mimic its postsynaptic actions reduced the occurrence and severity of AGS (81, 82). In DBA/2 mice at various ages, before, during, and after the period of maximal AGS, no changes in brain GABA concentration and glutamic acid decarboxylase (GAD) activity were found, which means that the susceptibility to AGS does not result from the reduced ability to synthesize and store GABA (83). Instead, lower binding sites for GABA in the brain of DBA/2 mice, compared with other non-susceptible strains, have been reported (84), and this reduction has been correlated with the age of susceptibility seizures (83). However, the affinities of these binding sites were found to be higher in DBA/2 mice than in control mice of the same age (85, 86). Studies on receptor binding, chloride flux, and response to GABA_A_ receptor drugs have suggested GABA_A_ receptor alterations in DBA/2 mice (53–55). In particular, a decreased density of benzodiazepine 3H-flunitrazepam binding sites in 28- to 29-day-old DBA/2 mice has been reported, suggesting a delayed development of these binding sites (85). Other studies have found an increase in benzodiazepine 3H-flunitrazepam binding sites in DBA/2 mice (at ~22 days of age) compared with age-matched C57BL/6J mice (87). For these reasons, the use of GABA_A_ agonists, GABA transaminase inhibitors, and some inhibitors of GABA reuptake may be useful in reducing the incidence and severity of AGS in DBA/2 mice (85, 88–90). An indirect involvement of GABA neurotransmission in AGS in DBA/2 has been also suggested by another study, in which gastrin-releasing peptide (GRP), a selective agonist for the BB2 subtype of bombesin receptor, was able to reduce AGS in audiogenic DBA/2 mice, probably through an increase of GABAergic function (91) (Figure 1).

In addition to neurotransmitters, synaptic hyperactivity and the sustained increase in circulating catecholamines are also involved in AGS and S-IRA; repeated induction of generalized seizures in DBA/2 mice can lead to Ca^2+^ overload and oxidative stress, which trigger mitochondrial dysfunction by generating reactive oxygen species (ROS) with consequent cardiomyocyte death (92–94).

## Efficacy studies of different drugs on AGS in DBA/2 mice

DBA/2 mice have been widely used not only as a genetic model of generalized reflex epilepsy but also for screening novel compounds to predict the clinical utility of novel treatments for drug-resistant epilepsy (78, 95).

All clinically used ASMs (carbamazepine, oxcarbazepine, felbamate, gabapentin, lamotrigine, phenytoin, phenobarbital, ethosuximide, levetiracetam, topiramate, valproate, brivaracetam, and perampanel) have shown efficacy against AGS in DBA/2 mice (9, 95–97) (Table 2).

**Table 2:** ED50 values (±95% confidence limits) of some ASMs against audiogenic seizures in DBA/2 mice.

**ASM**	**Wild running**	**Clonus**	**Tonus**	**References**
Carbamazepine	10.6 (8.1–13.8)	4.4 (3.6–5.4)	3.0 (2.6–3.8)	(98, 99)
Diazepam	0.49 (0.34–0.71)	0.28 (0.2–0.39)	0.24 (0.15–0.39)	(98, 99)
Ethosuximide	290 (207–406)	138 (96–198)	90 (70–116)	(98, 99)
Felbamate	114.6 (92–142.7)	48.8 (35–67)	23.1 (12.1–44)	(98, 99)
Gabapentin	38 (16–51)	20.3 (13.7–30.2)	13.9 (8.7–22.3)	(98, 99)
Gabapentin	38 (16–51)	20.3 (13.7–30.2)	13.9 (8.7–22.3)	(98, 99)
Lamotrigine	6.1 (4.6–8.1)	3.5 (2.4–5.1)	1.1 (0.7–1.8)	(98, 99)
Levetiracetam	15.38 (12.17–19.45)	9.77 (7.22–13.22)	7.89 (5.89–10.57)	(98, 99)
Oxcarbazepine	11.4 (9.7–13.3)	4.2 (3.0–5.88)	3.2 (2.7–3.79)	(98, 99)
Perampanel	ND	ND	0.47	(100)
Phenobarbital	7.1 (5.6–9.0)	3.4 (2.3–5.0)	2.4 (1.7–3.4)	(98, 99)
Phenytoin	4.3 (3.1–6.0)	2.5 (1.8–3.5)	2.0 (1.6–2.5)	(98, 99)
Retigabine	12.52 (9.92–15.8)	6.78 (4.60–9.99)	4.83 (2.98–7.83)	(98, 99)
Topiramate	22.9 (15.8–33.9)	12.12 (6.94–21.15)	6.12 (3.48–20.88)	(98, 99)
Valproate	84 (63–114)	43 (33–56)	31 (22–43)	(98, 99)
Brivaracetam	ND	2.4 (1.4–4.0)	ND	(101)
Unmodified CBDV BDS	127.56 (92. 39–176.12)	73.81 (53.24–102.34)	50.93 (29.85–86.88)	Unpublished data

Data are expressed in mg/kg. ND, not determined.

In addition, different studies have demonstrated the efficacy of classical benzodiazepines and their derivatives, 1,4- benzodiazepines and 1,5-benzodiazepines (82, 102). The different degree of anticonvulsant activity appears to be related to the benzodiazepine binding affinity (inhibition of [3H]flunitrazepam binding) (82, 89, 102). Compounds blocking the uptake of GABA (ethyl nipecotate and nipecotic acid) into neurons or glia and structurally diverse positive allosteric modulators of GABA_B_ receptors showed protection against AGS in DBA/2 mice (81, 103). Compounds that block excitatory neurotransmission by acting as antagonists at the NMDA and AMPA receptors showed the anticonvulsant effects in DBA/2 mice (75, 104).

Riluzole, a drug approved for the treatment of amyotrophic lateral sclerosis, was found to be protective against AGS in DBA/2 mice, with full protection against sound-induced tonic extension; this effect is reportedly due to the interaction of riluzole with glycine/NMDA and AMPA/kainate receptors (105).

Co-treatment of topiramate (TPM) with L-type Ca^2+^ channel modulators (nifedipine and Bay k 8644) or with AMPA/kainate receptor antagonists (NBQX and CFM) showed protection against tonic and clonic seizures in audiogenic mice (106).

The anticonvulsant effects of various other compounds, such as substances that enhance the 5-HT system (107), ligands for adenosine receptors (28, 69, 108), and carbapenem derivatives (109), have been also evaluated in DBA/2 mice. Treatment with cannabinoid receptor (CB1R) agonists has shown anticonvulsant effects against AGS, suggesting the involvement of these receptors in the susceptibility of DBA/2 mice to AGS (110). Similarly, cannabis-derived compounds, such as cannabidiol (CBD) and cannabidivarin (CBDV), have been shown to reduce AGS incidence, and the co-administration of CBD and CBDV showed synergic effects against AGS in audiogenic mice, blocking both clonic and tonic seizures through different mechanisms of CB1R (111, 112). Carbenoxolone, a chemical gap junction blocker, was shown to reduce the severity of AGS in DBA/2 mice, suggesting the role of connexins in epileptic seizures (113). Gap junctions play an important role in the neuronal network for both synchronized neuronal activity and field potential oscillations. During epileptic seizures and intercritical phases, electroencephalogram (EEG) signals and the degree of electrical coupling to the astrocytic gap junction were found to be correlated, contributing to the synchronization of the neuronal cell networks and inducing recurrent epileptiform activity (114). The antiepileptic effects of carbenoxolone could be due to its capacity to block both spontaneous bursts and epileptiform activity of neurons, although it does not do so directly. It seems to block the gap junction between astrocytes, suppressing their synchronization and supporting the possible direct involvement of the astrocytic gap junction in epileptiform activity and the indirect involvement in neurons (115). Among the different connexins (CX26, CX36, and CX32), CX43 has been found to affect epileptic seizures; its expression and electrical conduction have been found to increase after seizure, which can be reduced by carbenoxolone (114, 116).

The potential synergism of carbenoxolone with ASM, as well as the pharmacodynamic potentiation of ASM effects by statins, cannabinoid receptors agonists (98, 110), beta-adrenoceptor antagonists (117), and D-cycloserine, has been also studied in audiogenic DBA/2 mice (118). The potential anticonvulsant effects of a natural product, i.e., flavonoid-rich extract from orange juice (OJe), and its possible mechanism of action, were also studied on AGS-sensible DBA/2 (119). Finally, the protective effects of the new isoquinoline sulfonamides on AGS in DBA/2 mice were investigated by selectively inhibiting the isoforms of human carbonic anhydrase II and VII (hCA II and hCA VII) (120).

## DBA/2 mice as relevant models of human SUDEP

**Figure 2:**
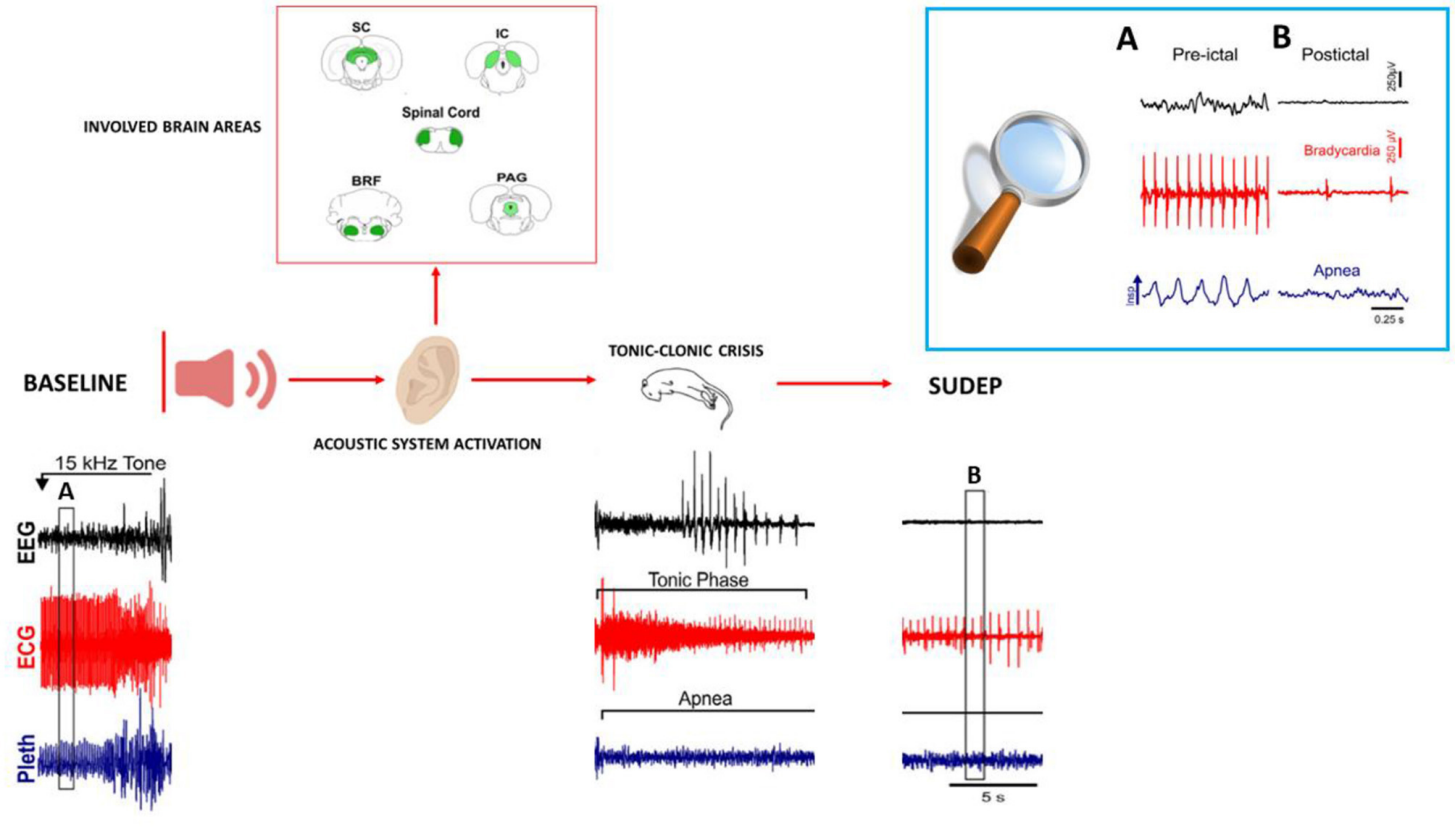
Representation of brain areas involved in AGS, and representative patterns of EEG (electroencephalogram), ECG (electrocardiogram) changes, and respiration (plethysmometer) before seizure onset, during the tonic–clonic phase up to SUDEP in audiogenic DBA/2 mice. The upper right box **(A, B)** shows the expanded traces from the corresponding boxes of the recordings shown below. All patterns are re-adapted by Wenker (123) in “Postictal death is associated with tonic phase apnea in a mouse model of sudden unexpected death in epilepsy.”

Animal models, in particular, genetically altered mice, develop spontaneous or induced seizures leading to death, and these models are able to show the role of autonomic dysregulation respiratory mechanisms that precede death. DBA/2 mice experience AGS when they are exposed to high-intensity acoustic stimulation, and these seizures are followed by a high occurrence of S-IRA, cardiac arrest, and sudden death, making these mice a valid model of human SUDEP (7, 121, 122) (Figure 2). In these mice, S-IRA, resulting from AGS, leads to subsequent death if not rapidly prevented by oxygen administration or mechanical ventilation, similar to epileptic patients (6, 26). Generalized seizures, particularly uncontrolled ones, are important risk factors for SUDEP because they propagate trans-synaptically or by spreading depression and interrupt the brainstem functions that control cardiorespiratory activity and its connections to cortical, diencephalic, and spinal cord areas. This causes RA with subsequent cardiac failure, mechanisms that are proposed as the main cause of human SUDEP (122, 124).

DBA/2 mice die in the tonic phase with hindlimb extension similar to human SUDEP with motor convulsive seizures (125). Death immediately following an AGS occurs with sequential events, including immediate respiratory failure followed by cardiac depression; this is observed in DBA/2 mice and is similarly observed in human SUDEP (24, 126). In DBA/2 mice, RA always precedes cardiac arrest, similar to terminal apnea, which is commonly responsible for human SUDEP. Therefore, altered brainstem function is linked with respiratory and cardiac dysfunctions, followed by terminal apnea and asystole, leading to SUDEP in both DBA/2 mice and epileptic patients.

The common cardiopulmonary events that contribute to death make DBA/2 mice an important model for evaluating the mechanisms of these events and translating this knowledge into the development of new strategies for SUDEP prevention.

The advantage of using DBA/2 mice as the SUDEP model is given by the predictability of seizures, allowing for the resuscitation of these mice and the repetition of tests and, consequently, the possibility to determine the mechanisms of RA (28).

In DBA/2 mice, AGS spreads to brainstem laryngomotor areas, causing laryngospasm and associated obstructive apnea with consequent death; the implantation of a surrogate airway protecting against death suggests that both laryngospasm and associated obstructive apnea are part of a common mechanism that leads to SUDEP in epileptic patients and murine species (24, 127). DBA/2 mice not only present many key features of human SUDEP, including autonomic dysregulation, apnea, hypoventilation, and cardiac arrhythmias, but also suggest the potential molecular mechanisms of SUDEP, such as modifications of serotonergic and adenosinergic neurotransmission, which significantly alter not only the seizure activity but also the brainstem respiratory network. 5-HT and adenosine show opposite effects on respiratory function (11, 28). Serotonergic neurons stimulate breathing, and the loss of brainstem serotonergic control of cardiorespiratory function induced by seizures can cause prolonged hypoventilation or apnea, contributing to SUDEP (128). In DBA/2 mice, the loss of serotonergic neurons reportedly induces S-IRA, the main factor for SUDEP (47), and the enhancement of 5-HT function in the brainstem with SSRI treatment reportedly reduces the occurrence of S-IRA (128). Similarly, dysregulation of serotonergic brainstem neurons, which regulate brainstem respiratory centers, and spreading of depolarization have been found in epileptic drug-resistant patients, and they have been correlated with an increased risk of human SUDEP (11, 25). Consequently, SSRI treatment has shown some protective effects by reducing the incidence of central apnea in SUDEP patients (129, 130). In addition, DBA/2 mice were found to exhibit diminished expression of 5-HT2C, 5-HT3, and 5-HT4 receptors in the rostral ventral medulla respiratory region; similar abnormalities of these 5-HT receptors expression were found in sudden infant death syndrome (131). Therefore, in addition to the evidence of the effect of serotonergic neurotransmission on seizure susceptibility, increases in 5-HT may positively affect seizure-related respiratory events associated with SUDEP in animal models and humans.

Furthermore, adenosine is also a possible contributor to SUDEP (49); excessive adenosine release and overactivation of adenosine receptors, induced by seizures, may give rise to excessive inhibition of brainstem respiratory centers; this causes lethal apnea or cardiac arrest, potentiating SUDEP through respiratory suppression in DBA/2 mice (28). The administration of caffeine, an adenosine receptor antagonist, reduced the incidence of seizure-induced RA, thus preventing SUDEP in audiogenic DBA/2 mice (28). Similarly, in different brain areas of epileptic patients, an altered adenosinergic system has been observed with the overactivation of A2A receptors, resulting in cardiorespiratory dysfunctions and increased risk of SUDEP (132). Consequently, the blockade of adenosine receptors should prevent SUDEP in human and experimental models (133, 134).

The translational research obtained in this experimental model has allowed us to identify that the serotonergic and adenosine neuronal networks of the brainstem may share a common final pathway with human SUDEP, and drugs that act on these neurotransmission systems may be potential targets for SUDEP prevention. Furthermore, the knowledge of cardiac and/or respiratory alterations due to AGS, which may contribute to sudden death in DBA/2 mice, indicates the translational potential of the use of these animals in clinical studies on SUDEP (135).

The elevated occurrence of death after AGS in DBA/2 mice suggests the existence of a genetic predisposition to respiratory depression, supporting the hypothesis of the presence of genetic predictors of SUDEP (40, 136).

Compared with other models, such as kainic acid (KA) or the pilocarpine animal model of status epilepticus (SE), the DBA/2 model has several advantages. One advantage of DBA/2 mice is that their death is not associated with SE, and they exhibit sudden death immediately after AGS. Their death is a result of RA, leading to subsequent cardiac failure, in agreement with the findings obtained in many cases of human SUDEP (3, 26). This distinct feature makes DBA/2 mice relevant SUDEP models. KA or pilocarpine-induced SE is useful as a model for human temporal lobe epilepsy (137), but these models do not induce SUDEP-like seizures. In fact, pilocarpine or KA injection produces SE in a healthy brain, a condition that is excluded from the current definition of SUDEP (2). Seizures appear in limbic and neocortical structures, but the seizure onset is believed to reside in the hippocampal formation with consequent hippocampal neurodegeneration and neuroinflammation, unlike what is observed in DBA/2 mice (138, 139).

Variability in SE induction and in the frequency and severity of spontaneous seizures, as well as variable mortality among different rodent strains, have also been found in SE animal models, causing difficulty not only in reproducing the TLE model but also in considering it a SUDEP model (140–142). Regarding the variability in the mechanisms of death, the mortality ranges from 5 to 30% in the SE KA-induced model (138), while in the pilocarpine model, mortality is much higher since ~30–40% of treated animals do not survive SE, and there are difficulties in SE induction due to pilocarpine-resistant animals (143, 144). Therefore, in these models, mice that died within 7 days from SE induction are believed to have failed to develop SE. Therefore, they are not considered to have died of SUDEP since they likely did not develop spontaneous seizures. Unlike audiogenic DBA/2 mice in which seizure-induced respiratory depression leads to sudden death, some of these animals can show small seizure activity, some can also show transient apnea, and others can show respiratory depression. Therefore, it is not possible to understand which of these behaviors are related to SUDEP manifestation. Additionally, in these animal models of SE, significant changes in cardiac structure and function are found after the SE period (145).

Some studies reported obstructive apnea, cardiac dysfunction, and death as a response to KA-induced seizure (146), while other studies showed that some mice, survived by pilocarpine-induced SE, died suddenly after several weeks when they developed spontaneous seizures (147, 148). However, these responses to KA or pilocarpine-induced SE are different from mechanisms that cause respiratory depression and death in the DBA/2 model. In addition, genetic mutations together with neurotransmission alterations can explain the higher susceptibility to AGS and the consequent death of DBA/2 mice.

Although this audiogenic model more strongly resembles human SUDEP in many respects, DBA/2 mice present some limitations to achieving a full comprehension of respiratory dysfunction in chronic epilepsy and SUDEP, which is because AGS and S-IRA are induced by intense acoustic stimulation, and these mice do not develop spontaneous recurrent seizures unlike what occurs in human SUDEP, which in any case results from spontaneous seizures (28). Furthermore, in DBA/2 mice, there is a high incidence of death after AGSs, while the probability of sudden death after an epileptic seizure is lower in patients (5). In particular, in DBA/2 mice, if AGS-induced fatal RA is not prevented, they die (11), whereas epileptic patients who die of SUDEP can have numerous seizures before the fatal event occurs (1).

In addition, the susceptibility to AGS of DBA/2 mice seems to be age-dependent, being high in young mice (21–28 days postnatal) and decreasing with age. The young age at which DBA/2 mice show susceptibility to AGS could be a limitation in transferring the results to human settings, particularly in investigating any chronic treatment, which would be needed for human SUDEP prevention. For this, DBA/2 mice may be appropriate as an acute mouse model of SUDEP. However, the young age at epilepsy onset and the duration of the seizure disorder, which often occurs during childhood, are the main risks of human SUDEP (17). In particular, the risk of SUDEP is greater for patients with childhood-onset epilepsy that is chronic and persists into adulthood than for patients with epileptic seizures that appear in adulthood. The clinical implication of this age-dependent susceptibility to seizure may be related to the higher incidence of sudden death in children with severe forms of epilepsy than in adults. However, recent studies in children have documented a similar incidence to that of adults (21). Despite these differences, studies of sudden death in young AGS-susceptible mice may be useful for increasing the knowledge of complex pathophysiologic mechanisms underlying human SUDEP and for assessing potential therapies to prevent SUDEP.

## Conclusion

In epilepsy, SUDEP is a major clinical problem for epileptic patients and their families. Although different studies have identified numerous risk factors for SUDEP, we are still unable to prevent or diminish the incidence of SUDEP. Furthermore, although adherence patterns in ASM can limit seizures in most patients, they do not alter long-term prognosis or prevent epilepsy progression or the risk of SUDEP. The high mortality rate associated with certain types of epilepsy points to respiratory depression, decreased heart frequency, autonomic dysfunction, and altered neurotransmitter systems in the brainstem. Although treatments such as SSRIs and adenosine antagonists have been experimented with, their clinical application remains limited.

Studies in animal models of epilepsy are essential to help understand the pathophysiological mechanisms associated with seizures and SUDEP. In particular, DBA/2 strains are inbred mice prone to AGS-induced fatal RA and frequently used as SUDEP models. These mice not only summarize numerous key features of human SUDEP, including altered cardiorespiratory function and pulmonary impairment following audiogenic seizures, but also allow for accurate investigations of molecular mechanisms present in SUDEP, such as serotonergic and adenosinergic dysfunctions and genetic susceptibility. Brainstem neurotransmitters may provide new targets for interventions to prevent premature death from SUDEP.

Translation of basic experimental research into human epilepsy could be represented by DBA/2 mice, whose response to ASM has permitted the development of new medications and therapy trials.

However, in this experimental model, SUDEP susceptibility is related to acoustic stress only at a young age. Therefore, DBA/2 mice could represent only an acute SUDEP model. However, given the translational value of the knowledge that can emerge from using this animal model, it would be advisable to carry out further and more specific studies.

## Author contributions

FB, LGa, and AL conceptualized the manuscript framework, performed literature research, and drafted the manuscript. MT, LGu, and VR were also involved in drafting the manuscript and creating the tables. RC and GD critically reviewed the manuscript. All authors reviewed the drafted manuscript for critical content and approved the final version.
